# Magnetic Solid-phase Extraction with Fe_3_O_4_/Molecularly Imprinted Polymers Modified by Deep Eutectic Solvents and Ionic Liquids for the Rapid Purification of Alkaloid Isomers (Theobromine and Theophylline) from *Green Tea*

**DOI:** 10.3390/molecules22071061

**Published:** 2017-06-25

**Authors:** Guizhen Li, Xiaoqin Wang, Kyung Ho Row

**Affiliations:** Department of Chemistry and Chemical Engineering, Inha University, Incheon 402751, Korea; 22162368@inha.edu (G.L.); 22161803@inha.edu (X.W.)

**Keywords:** deep eutectic solvents, ionic liquids, Fe_3_O_4_/molecularly imprinted polymers, solid-phase extraction

## Abstract

Different kinds of deep eutectic solvents (DES) based on choline chloride (ChCl) and ionic liquids (ILs) based on 1-methylimidazole were used to modify Fe_3_O_4_/molecularly imprinted polymers (Fe_3_O_4_/MIPs), and the resulting materials were applied for the rapid purification of alkaloid isomers (theobromine and theophylline) from *green tea* with magnetic solid-phase extraction (M-SPE). The M-SPE procedure was optimized using the response surface methodology (RSM) to analyze the maximum conditions. The materials were characterized by Fourier transform infrared spectroscopy (FI-IR) and field emission scanning electron microscopy (FE-SEM). Compared to the ILs-Fe_3_O_4_/MIPs, the DESs-Fe_3_O_4_/MIPs were developed for the stronger recognition and higher recoveries of the isomers (theophylline and theobromine) from *green tea*, particularly DES-7-Fe_3_O_4_/MIPs. With RSM, the optimal recovery condition for theobromine and theophylline in the M-SPE were observed with ratio of methanol (80%) as the washing solution, methanol/acetic acid (HAc) (8:2) as the eluent at pH 3, and an eluent volume of 4 mL. The practical recoveries of theobromine and theophylline in green tea were 92.27% and 87.51%, respectively, with a corresponding actual extraction amount of 4.87 mg·g^−1^ and 5.07 mg·g^−1^. Overall, the proposed approach with the high affinity of Fe_3_O_4_/MIPs might offer a novel method for the purification of complex isomer samples.

## 1. Introduction

*Green tea* has many beneficial effects, such as reducing the risk of cancer and cardiovascular diseases, antioxidant effects, anti-inflammation, and anti-obesity. These effects are due mainly to its purine alkaloids, such as methylxanthines [1,2]. Theobromine (3,7-dimethylxanthine) and theophylline (1,3-dimethylxanthine) are common alkaloid isomers that belong to the methylxanthine family, which is most abundant in food [3,4]. The traditional approaches to determining the two methylxanthines have some disadvantages, such as being time-consuming and tedious, low sensitivity, and consuming large amounts of toxic organic solvents [5,6]. Owing to the complexity of the sample matrices, sample pretreatment is a crucial step in the analytical method. Thus far, the most widely used sample pretreatment method is solid-phase extraction (SPE) [7]. On the other hand, SPE is limited when the concentrations of the target compounds are extremely low or there is some interference of the complex in biological samples. Compared to traditional SPE, magnetic SPE (M-SPE) can be used effectively for the purification of trace amounts of the targets [8,9].

Magnetic materials as sorbents have been observed with several advantages over traditional sorbents [10,11,12,13]. M-SPE is a new procedure for the preconcentration of target analytes from large volumes based on the use of magnetic or magnetizable adsorbents [14]. The separation process can be performed directly in a sample solution containing a solid sorbent, and the magnetic materials can be collected and separated from the liquid phase using a magnetic field, which avoids a tedious filtration or centrifugation procedure [15,16]. Fe_3_O_4_ materials, as magnetic materials, could be isolated readily from sample solutions through the application of an external magnetic field, for example, methods used in capillarity by magnetic fields like magnetic in tube solid phase microextraction (Magnetic-IT-SPME) provided a enhancement of the extraction efficiency for triazines [17,18]. This rapid separation technique has been applied successfully to the separation and enrichment of peptides, proteins, pharmaceutical residues, nucleic acids, and several other substances in biological samples [19,20,21]. Moreover, the specific characteristics of these particles that make them widely applicable in many other fields include excellent magnetic responsiveness, high dispersibility, and ease of surface modification [22].

Deep eutectic solvents (DESs) are usually composed of an organic salt (such as choline chloride (ChCl) and choline acetate) and a hydrogen-bond donor compound (such as amides, amines, alcohols and carboxylic acids) [23], which have attracted considerable attention as a modification of materials, such as electrocatalysts, extraction media, and separation [24,25,26,27]. Ionic liquids (ILs) consisting of imidazolium cations and different counteranions, have also attracted increasing interest as a modification to nanoparticles or polymerized materials for a wide range of synthesis, catalysis, electrochemistry, and solid-phase extraction [28,29,30,31]. Owing to these excellent properties, DESs and ILs have been applied for hydrogen bonding to a material surface, resulting in improved adsorption performance. 

The Box-Behnken design (BBD) is a type of Response Surface Methodology (RSM), which explores the relationships between several explanatory variables and one or more response variables [32]. It has been applied successfully to analyze the conditions in food and pharmaceutical research [33,34]. The main idea of the RSM is to use a sequence of designed experiments to obtain an optimal response [35,36], and it is helpful for finding the optimal condition of nanoparticles applied to M-SPE using RSM.

In this study, DESs and ILs were used to modify the surface of Fe_3_O_4_/MIPs to obtain DESs (ILs)-Fe_3_O_4_/MIPs, and the resulting nanoparticles were applied to the rapid purification of alkaloid isomers (theobromine and theophylline) from *green tea* with M-SPE. The M-SPE procedure was optimized with RSM to determine the maximum condition. Structural information about the Fe_3_O_4_/MIPs and DESs (ILs)-Fe_3_O_4_/MIPs was obtained by Fourier transform infrared (FTIR) spectroscopy, and the selective recognition ability was investigated by adsorption experiments. 

## 2. Results and Discussion

### 2.1. Validation of MSPE-HPLC Method

DESs-Fe_3_O_4_/MIPs and ILs-Fe_3_O_4_/MIPs as the M-SPE material for the purification of theobromine and theophylline were studied, and validation of the method under the optimized protocols was achieved. The calibration curve for theobromine and theophylline was in the range, 5–100.0 μg mL^−1^. The regression equation was *Y* = *1.3283 × 10^4^ X* + *9.1044 × 10^4^* (*R^2^*= 0.9993), and *Y* = *6.1173 × 10^4^ X − 1.0309 × 10^4^* (*R^2^*= 0.9999), respectively (Table 1). The method recoveries ranged from 89.96%–96.74% for theobromine, and 87.72%–95.79% for theophylline when the concentrations were 10.0, 50.0, and 100.0 μg·mL^−1^ at the three levels. The RSD (relative standard deviation) of the intra-day and inter-day determination was less than 4.19% (Table 2).

### 2.2. Purification of the Extracts with Solid-Phase Extraction

**Figure 1:**
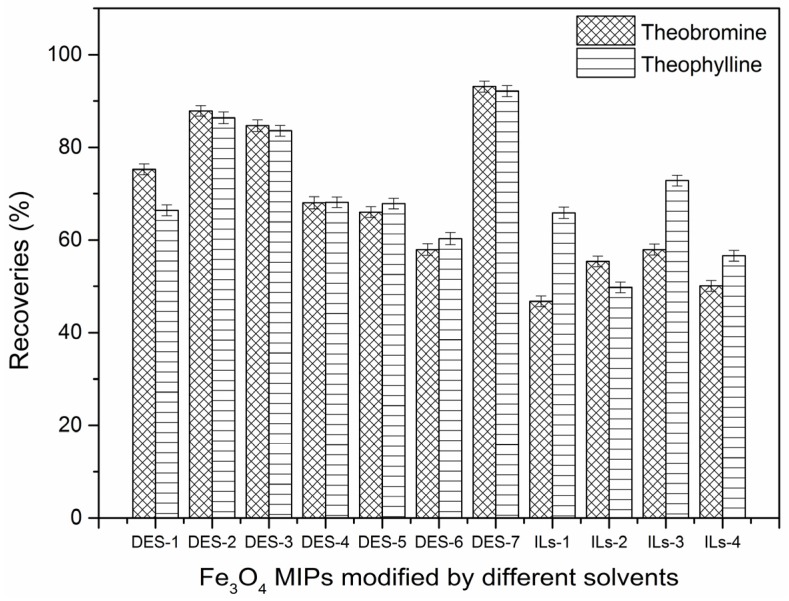
The recoveries of theobromine, and theophylline by different kinds of Fe_3_O_4_/MIPs.

The recoveries of the extracted samples with DESs-Fe_3_O_4_/MIPs and ILs-Fe_3_O_4_/MIPs indicated that they had high selectivity and affinity for the analytes in an aqueous environment. Compared to the ILs-Fe_3_O_4_/MIPs, DESs-Fe_3_O_4_/MIPs had a better effect. The method was reliable and could be used for the trace analysis of theobromine and theophylline in green tea. In particular, DES-7-Fe_3_O_4_/MIPs were observed with the highest recoveries and recognition towards theobromine and theophylline in green tea. Figure 1 presents the recoveries of theobromine and theophylline by the different types of Fe_3_O_4_/MIPs.

DES-7, the type of functional synthetic molten salt composed of an organic cation (ChCl) and anion, was introduced in the procedure of Fe_3_O_4_/MIPs synthesis to improve the affinity and selectivity of the resulting nanoparticles. The electrostatic and ion-exchange interactions of the DES-7 acylamino group was firmly embedded in the Fe_3_O_4_/MIPs, while -NH of choline chloride and urea could be the functional group on the surface of the nanoparticles to form a hydrogen bond with the template, which was beneficial to the resolution of complex samples comprising the components with a wide range of polarities. 

In addition, excessive DES-7 could be linked to the surface of the materials because an auxiliary solvent can also promote the functional solvents to form the specific binding sites and make Fe_3_O_4_/MIPs more rigid without shrinking or swelling, which can also improve the affinity and selectivity of the Fe_3_O_4_/MIPs. 

### 2.3. Characterizations

**Figure 2:**
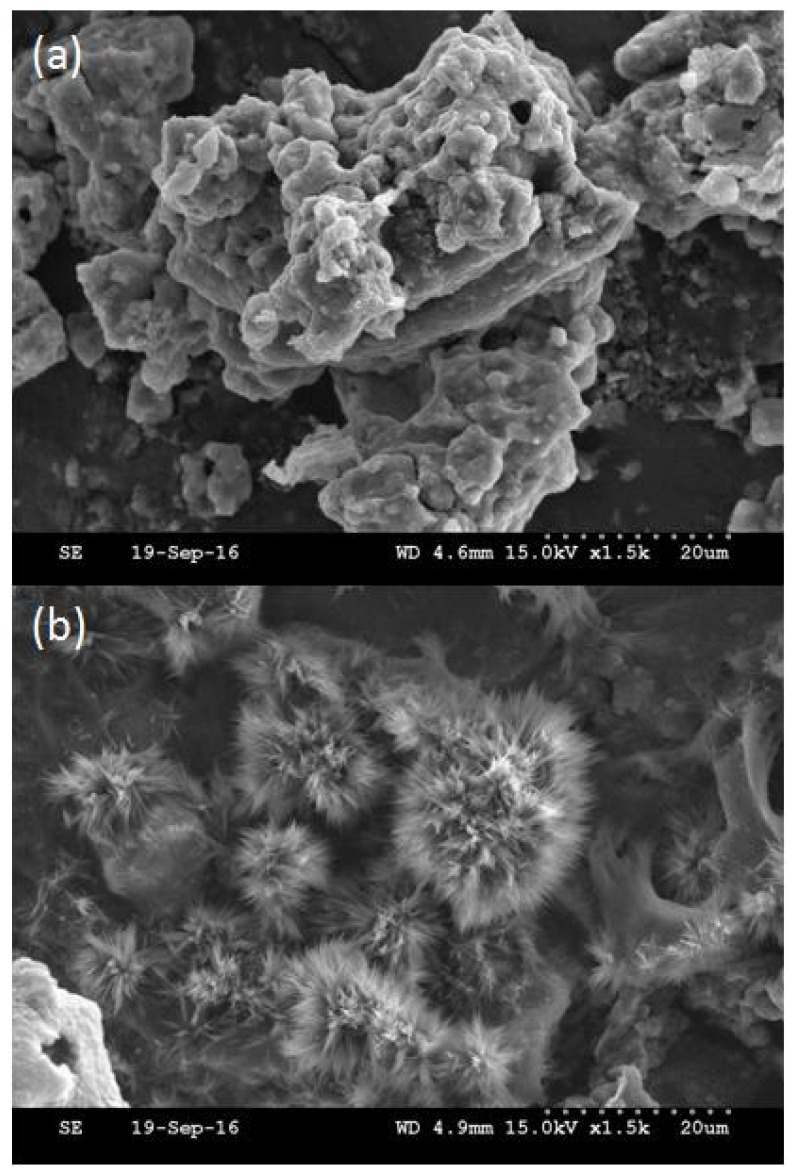
FE-SEM images of Fe_3_O_4_/MIPs (**a**) and DES-7-Fe_3_O_4_/MIPs (**b**).

Figure 2 shows many macropores and flow-through channels inlaid in the network skeletons of the Fe_3_O_4_/MIPs and DESs-7-Fe_3_O_4_/MIPs. In addition, compared to the Fe_3_O_4_/MIPs, the surface of the DESs-7-Fe_3_O_4_/MIPs was irregular and agglomeration was observed. The rough surface of the DESs-7-Fe_3_O_4_/MIPs was porous, which corresponded to the decomposition of the magnetic microsphere surface. The looser skeleton not only decreased the mass-transfer resistance but also increased the surface area of the nanoparticles in favor of embedding theobromine and theophylline into the cavity of the DESs-7-Fe_3_O_4_/MIPs, which resulted in easier adsorption and desorption and higher stability and reproducibility.

**Figure 3:**
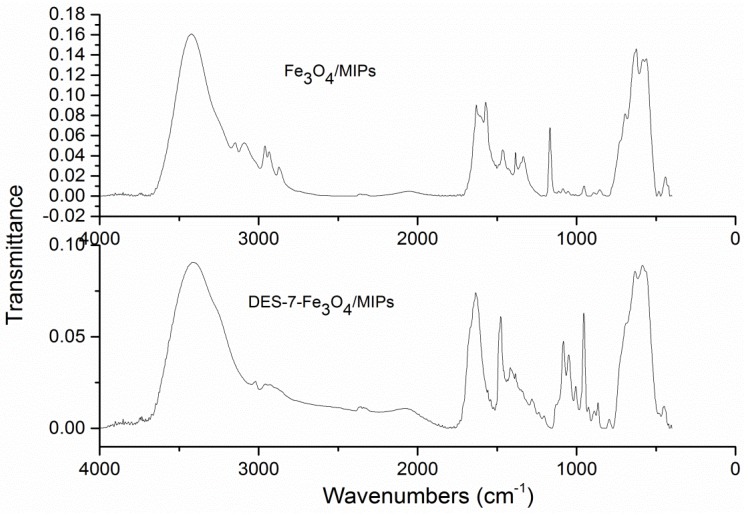
FT-IR images of Fe_3_O_4_/MIPs and DES-7-Fe_3_O_4_/MIPs.

The FT-IR spectra of the Fe_3_O_4_/MIPs and DESs-Fe_3_O_4_/MIPs (Figure 3) showed a great difference in the fingerprint region. Fe_3_O_4_/MIPs displayed the characteristic peaks of Fe-O at 568 cm^−1^, while the DESs-7-Fe_3_O_4_/MIPs exhibited the relatively strong band of the -OH group at 1560 cm^−1^ and 3320 cm^−1^. In addition, the band at approximately 2365 cm^−1^ was assigned to the stretching vibration of -CH_2_ or -CH_3_ from choline chloride; the band at 1340 cm^−1^ was assigned to absorption by the C-O group; the band at 2365 cm^−1^ was ascribed to the stretching vibrations of the tertiary amine group (C-N) from choline chloride. Compared to Fe_3_O_4_/MIPs, FT-IR spectra of DESs-7-Fe_3_O_4_/MIPs showed that the hydrogen bond was strengthened and enhanced.

### 2.4. Adsorption Properties of Fe_3_O_4_/MIPs and DESs-7-Fe_3_O_4_/MIPs 

**Figure 4:**
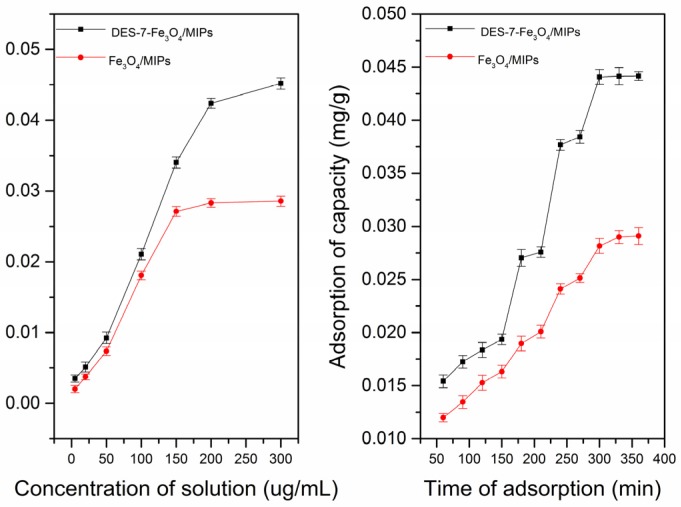
The static and dynamic adsorption capacity curves of two materials (Fe_3_O_4_/MIPs, and DES-7-Fe_3_O_4_/MIPs) for Theobromine.

**Figure 5:**
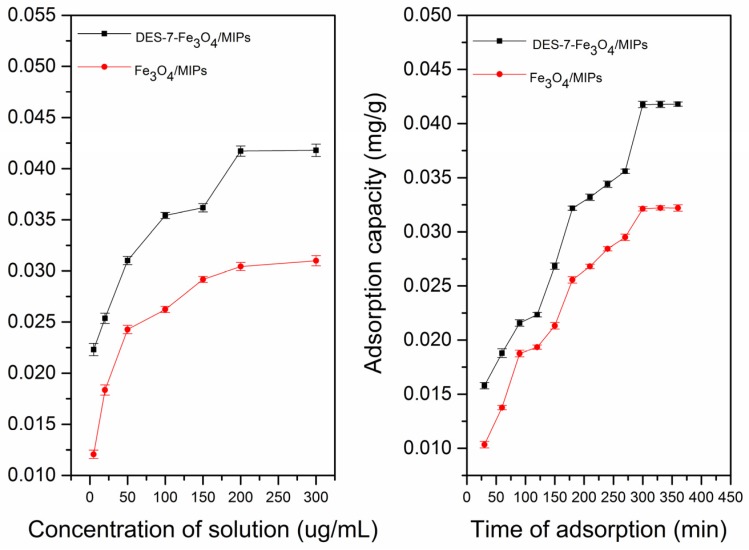
The static and dynamic adsorption capacity curves of two materials (Fe_3_O_4_/MIPs, and DES-7-Fe_3_O_4_/MIPs) for Theophlline.

The static and dynamic adsorption curves were used to evaluate the binding property of the Fe_3_O_4_/MIPs and DES-7-Fe_3_O_4_/MIPs at room temperature. The static and dynamic adsorption experiments (Figure 4 and Figure 5, respectively) showed that the amounts of theobromine and theophylline sorbed by the nanoparticles increased with increasing concentration (5.00–300.00 μg·mL^−1^), and DES-7-Fe_3_O_4_/MIPs displayed higher affinity than Fe_3_O_4_/MIPs. The difference in the adsorption capacity between the Fe_3_O_4_/MIPs and DES-7-Fe_3_O_4_/MIPs increased with increasing theobromine and theophylline concentration until both reached equilibrium. The static adsorption capacity of the analytes on the DES-7-Fe_3_O_4_/MIPs (0.0441 mg/g for theobromine, and 0.0418 mg/g for theophylline) revealed better affinity than the Fe_3_O_4_/nanoparticles. 

Moreover, the dynamic adsorption capacity of the analytes of the DES-7-Fe_3_O_4_/MIPs displayed better affinity than the Fe_3_O_4_/MIPs. Because of the different spatial structures of the two analytes, they were studied to reach adsorption equilibrium at different times (270 min for theobromine and 300 min for theophylline). Having specific recognition sites for theobromine and theophylline, the DES-7-Fe_3_O_4_/MIPs showed good sensitivity for the two targets.

### 2.5. Optimization of M-SPE Procedure

**Figure 6:**
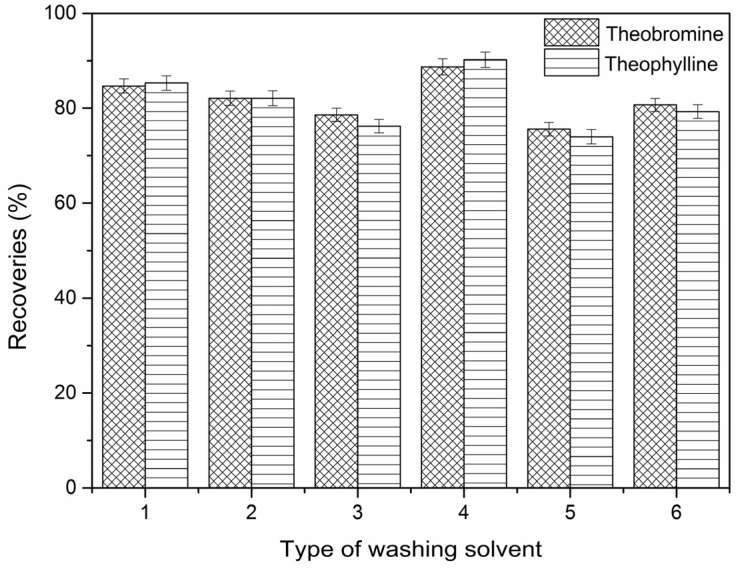
Effect of washing solvents for theobromine, and theophyline (1: MeCN; 2: EtOH; 3: H_2_O; 4: MeOH; 5: EtOAc; 6: Acetone).

Among the materials, the DES-7-Fe_3_O_4_/MIPs showed the best recognition for the two targets. Figure 6 shows the effects of MeCN, EtOH, H_2_O, MeOH, EtOAc, and acetone, respectively, as the washing solution in the M-SPE procedure with DES-7-Fe_3_O_4_/MIPs. Methanol was observed as the best washing solution among the five types of washing solution.

**Figure 7:**
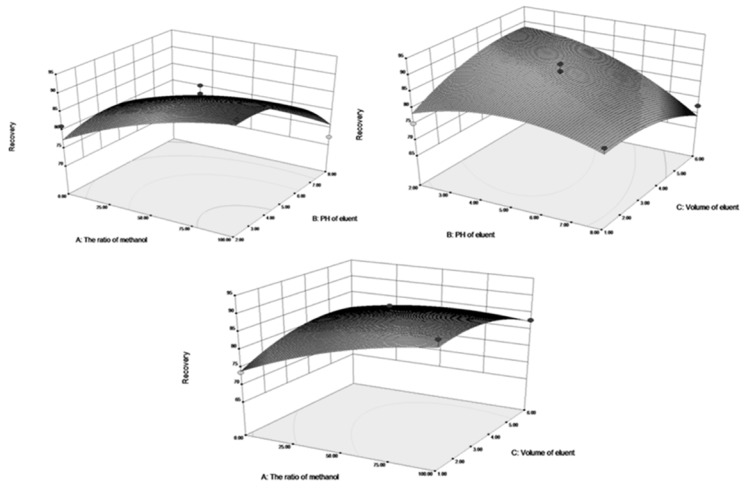
Effects of ratio of metanol, PH of eluent, volume ofeluent, and their reciprocal 3D response interaction on recoveries for theobromine with DES-7-Fe_3_O_4_/MIPs in SPE.

**Figure 8:**
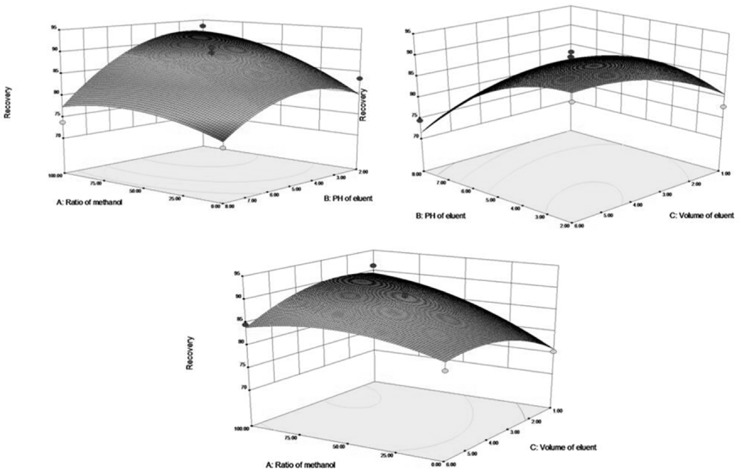
Effects of ratio of metanol, PH of eluent, volume ofeluent, and their reciprocal 3D response interaction on recoveries for Theophlline with DES-7-Fe_3_O_4_/MIPs in SPE.

Figure 7 shows the 3D response surface plots. In this figure, the recoveries of theobromine increased when ratio of methanol as the washing solution was in the designed range from 0:1 to 100:0, and the response increased initially then decreased when the pH of the eluent was increased from 2 to 8, and there was a small increase followed by a decrease as the eluent volume ranged from 1 mL to 6 mL. Moreover, the recoveries of theophylline (Figure 8) showed similar changes. 

The recoveries of theobromine and theophylline decreased with decreasing ratio of ethanol. As the ratio of ethanol decreased, the polarity of the washing solvents decreased and in this way, the interference of the impurities in the matrix of samples and materials were reduced. The different pH values were attributed to the destruction of the hydrogen-bonding interaction between the template molecules and imprinted cavities. The eluent volume is an important parameter that influences the recoveries of theophylline and theobromine in the M-SPE procedure. 

The optimal recoveries condition for theobromine and theophylline were observed under the same condition (ratio of methanol in water (80%), pH of the eluent (PH = 3), and the eluent volume (4 mL) and was estimated using the model equation by solving the regression equation and analyzing the response surface contour plots. The theoretical recoveries of theobromine and theophylline under the above conditions were 92.27% and 87.51%, respectively. 

### 2.6. Analysis of Green Tea Samples

**Figure 9:**
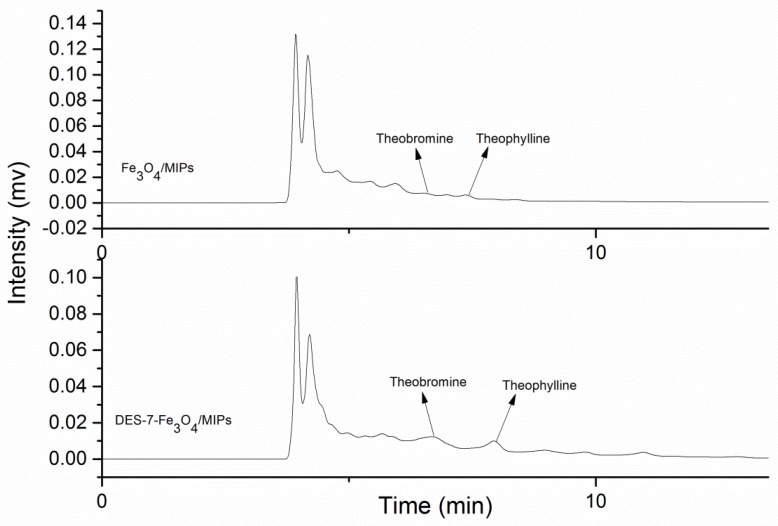
The purification chromatograms of green tea extracts with two materials (Fe_3_O_4_/MIPs, and DES-7-Fe_3_O_4_/MIPs) by M-SPE. (Column: C_18_ column, mobile phase: methanol-water-acetic acid (20/80/2, *v/v/v*), flow rate: 0.8 mL·min^−1^, UV: 280 nm, injection: 10 μL).

The proposed M-SPE method was used to determine the alkaloid isomers (theobromine and theophylline) in *green tea* under the optimal conditions. The optimal conditions for the recovery of theobromine and theophylline were ratio of methanol (80%) as the washing solution, methanol/HAc (8:2) eluent at pH 3, and an eluent volume of 4 mL. The practical recoveries of theobromine and theophylline in *green tea* were 92.27% and 87.51%, with corresponding extraction amounts of 4.87 mg·g^−1^ and 5.07 mg·g^−1^. Figure 9 presents chromatograms of the sample extracts using Fe_3_O_4_/MIPs and DES-7-Fe_3_O_4_/MIPs. Compared to the two chromatograms, the chromatogram of the DES-7-Fe_3_O_4_/MIPs with fewer interfering peaks and a better chromatogram shape, and the peaks of the two isomers were easier to distinguish.

## 3. Experimental

### 3.1. Reagents and Materials

*Green tea* was purchased from a local market (Incheon, Korea). Theophylline and theobromine were obtained from Sigma-Aldrich. Co, Ltd. (Spruce, St. Louis, MO, USA). Choline chloride (ChCl), ethyl acetate (EtOAc), acetonitrile (MeCN), and acetone were obtained from Daejung Chemicals & Metals Co., Ltd. (Siheung-do, Korea). 1-Methylimidazole was supplied by Sigma-Aldrich. Co, Ltd. (Munich, Germany). Ethylene glycol, glycerol, 1,4-Butanediol and urea were acquired from Duskan Pharmaceutical Co. Ltd. (Seoul, Korea). Iron trichloride hexahydrate (FeCl_3_·6H_2_O) and iron dichloride tetrahydrate (FeCl_2_·4H_2_O) were purchased from Sigma-Aldrich. Co, Ltd. Acetic acid, formic acid, and propionic acid were bought from Duskan Pure Chemical Co., Ltd. Methanol and ethanol were obtained from Fisher Scientific Co., Ltd. (Seoul, Korea). All other solvents used in the experiment were of HPLC or analytical grade.

### 3.2. Chromatography and Sample Pretreatment

The chromatography system consisted of a Waters 600 s Multi solvent Delivery System, Waters 1515 liquid chromatography pump (Waters, MA, USA), a Rheodyne injector (20 μL sample loop) and a variable wavelength 2489 UV dual channel detector. EmpowerTM 3 software (Waters) was used as the data acquisition system. The analysis was performed on an OptimaPak C_18_ column (5 μm, 250 × 4.6 mm, i.d., RStech Corporation, Daejeon, Korea). The mobile phase was methanol-water-acetic acid (20/80/2, *v/v/v*). The flow rate was 0.8 mL·min^−1^, and the detection wavelength was 280 nm.

The standard theobromine and theophylline were dissolved in methanol to a concentration of 1000.00 μg·mL^−1^. For method development, a series of standard solutions containing theobromine and theophylline were prepared at five concentrations ranging from 5.00–100.00 μg·mL^−1^. The standard curve of theobromine and theophylline were linear by assaying five data points and measured twice. 

### 3.3. Preparation of Fe_3_O_4_/MIPs and DESs (ILs)-Fe_3_O_4_/MIPs

#### 3.3.1. Preparation of DESs and ILs

The DESs were formed by ChCl and ethylene glycol, glycerol, and 1,4-butanediol, urea, formic acid, acetic acid, and propionic acid (1/2, *n/n*) in a conical flask, and heated to 80 °C with constant stirring for 2 h until a homogeneous liquid formed. Table 3 lists the DESs.

The ILs were formed with 1-methylimidazole (1 mL) and an excess of bromoethane, bromobutane, bromohexane, and bromohexane in a round-bottom flask at 80 °C for 6 h and washed with ethyl acetate after cooling to room temperature. The resulting [EMIM][Br], [BMIM][Br], [HMIM][Br], and [OMIM][Br] were produced. Table 4 lists the ILs.

#### 3.3.2. Preparation of Fe_3_O_4_/MIPs 

The Fe_3_O_4_/MIPs were prepared by a chemical coprecipitation method [37,38]. FeCl_2_·4H_2_O (6.0 g), FeCl_3_·6H_2_O (15.6 g), and hydrochloric acid (12 M, 2.55 mL) were dissolved in pure water (50 mL). The mixture was added dropwise to a NaOH solution (250 mL, 1.5 M) with vigorous stirring with nitrogen gas passing continuously through the solution during the reaction. Subsequently, the magnetic precipitates were isolated from the solution using a magnet, and washed sequentially with water and ethanol before being dried at 50 °C in a vacuum for 24 h.

#### 3.3.3. Preparation of DESs (ILs)-Fe_3_O_4_/MIPs

A 200 mg sample of Fe_3_O_4_/MIPs was added to a 250 mL round-bottom flask and dispersed ultrasonically for 20 min. Subsequently, isopropanol (8 mL) with the DESs (ILs, 2 mL) was added to the flask and reacted at 80 °C by stirring these two components for 24 h. After the reaction, the product was separated from the reaction medium under an applied magnetic field, rinsed three times with pure water, twice with isopropanol, and then separated using an external magnetic field. The product was then dried in the vacuum.

MAA was each added to Fe_3_O_4_ particles in a clean, dry round bottomed flask containing a magnetic stirring bar. Template molecules of theobromine and theophylline, were then allowed to form hydrogen bonds between them. The emulsions were sonicated for 20 min and stored at 4 °C in the dark. EDMA (20 mmol) and AIBN (1.0 mmol) were added to the mixed solutions together at 60 °C for 12 h. After polymerization, the bulk polymers were ground and sieved through a 105 μm stainless-steel mesh. The resulting polymers were washed with MeOH–HOAc (9:1, *v/v*) in a Soxhlet apparatus to remove the templates, and dried in a vacuum for 12 h. In the same case as the other synthetic processes, deep eutectic solvents-polymers without templates (DESs-Fe_3_O_4_/NIPs) were prepared in the absence of DESs. The magnetic polymer without templates (Fe_3_O_4_/NIPs) was also synthesized using the same procedure but in the absence of the templates and without DESs. 

### 3.4. Characterization of the Fe_3_O_4_/MIPs and DESs (ILs)-Fe_3_O_4_/MIPs

The Fe_3_O_4_/MIPs and DESs (ILs)*-*Fe_3_O_4_/MIPs were dried at 50 °C for 24 h. The amount of the obtained materials was ground together with KBr for tablets. The spectra were then analyzed using a Fourier transform infrared (FTIR) spectrometer (VERTEX 80V) in the wave number range of 400–4000 cm^−1^.

The morphological microstructures of these materials were observed by field emission scanning electron microscopy (FE-SEM, SE-4200, MERLIN Compact, ZEISS, Jena, Germany).

### 3.5. Absorption Capacity of Fe_3_O_4_/MIPs and DESs-Fe_3_O_4_/MIPs

At room temperature, for the static absorption experiment, 20.0 mg each of the proposed materials was mixed with 2.0 mL of the theobromine and theophylline standard solutions (5.0–300.0 μg·mL^−1^) in centrifuge tubes. After shaking for 8 h, the mixture was centrifuged, and the theobromine and theophylline concentrations in the upper solution were measured to calculate the adsorption capacities. 

For the dynamic adsorption experiment, 1.0 mL of the theobromine and theophylline standard solution (150.0 μg·mL^−1^) was mixed with 10.0 mg each of the proposed nanoparticles and shaken for 30–360 min. After centrifuging, the theobromine and theophylline levels in the upper solution for various times were determined to calculate the adsorption capacities.

The adsorption quantity (Q) was calculated based on the change in the free concentration (C *_free_*) and the initial concentration (C_0_) of the template by Equation (1), where V is the volume of the solution and W is the mass of the material powder:

(1) $$Q=\frac{(C_{0}-C_{free})\times V}{W}$$

### 3.6. Purification of Theobromine and Theophylline from Green Tea by M-SPE 

*Green tea* was dried in an oven at 50 °C and ground to a powder. The designated conditions were an ultrasonic time of 1 h, an absolute ethyl alcohol as the extracting solution, and the ratio of material to liquid ratio of 1:20 (g·mL^−1^). The suspension was then filtered to obtain the extraction samples. After each SPE cartridge was preconditioned sequentially by methanol (1.5 mL) and deionized water (1.5 mL) to clean the cartridge, 1.0 mL of the extract solution was loaded on the cartridge followed by 1.5 mL of MeCN, EtOH, H_2_O, MeOH, EtOAc, and acetone, respectively, as the washing solution and methanol/HOAc (8:2) (1.5 mL) mixture solution, as the commonly used elution in SPE was chosen as the elution solution. The effluents at every step were collected using a 1.0 mL syringe, which was connected to the bottom of the SPE cartridge to ensure a suitable and constant flow rate.

### 3.7. Optimization of M-SPE Procedure

To achieve the excellent extraction efficiency, several parameters involving washing solvent, the pH of the eluent solvent and elution volume were optimized, and the 17-run BBD was applied to optimize the procedure statistically. In Table 5, these three factors were designated as *X_1_*, *X_2_*, and *X_3_* prescribed into three levels, coded +1, 0, and −1 for high, intermediate, and low values, respectively. The three test variables were coded according to the following equation:

(2) $$X_{i}=\frac{X_{i}-X_{0}}{\Delta X}\;i=1,2,3$$

In this equation, *x_i_* is the coded value of the independent variable, *Xi* is the actual value of the independent variable, *X_0_* is the actual value of the independent variable at the center point, and △X is the step change value of the independent variable. A second-order polynomial model was fitted to correlate the relationship between the independent variables and the response (target recovery) to predict the optimized conditions:

(3) $$Y=A_{0}+\sum_{i=1}^{3}A_{i}X_{i}+\sum_{i=1}^{3}A_{ii}X_{i}^{2}+\sum_{i=1}^{2}\sum_{j=1+1}^{3}A_{ij}X_{i}X_{j}$$

In this equation, *Y* is the dependent variable, *A_0_* is a constant, and *A_i_, A_ii_*, and *A_ij_* are coefficients estimated by the model. *X_i_* and *X_j_* are the levels of the independent variables that represent the linear, quadratic, and cross-product effects of the *X_1_*, *X_2_*, and *X_3_* factors on the response, respectively. The model evaluated the effects of each independent variable on the response. 

## 4. Conclusions

Different types of DESs based on choline chloride and ILs based on 1-methylimidazole were used to modify Fe_3_O_4_/MIPs. The resulting nanoparticles were applied to the rapid purification of alkaloid isomers (theobromine and theophylline) from *green tea* by M-SPE. The M-SPE procedure was optimized by RSM to determine the best conditions. The materials were characterized by FT-IR spectroscopy and FE-SEM. Compared to the ILs-Fe_3_O_4_/MIPs, the DESs-Fe_3_O_4_/MIPs were developed for stronger recognition and higher recoveries of theophylline and theobromine from green tea, particularly DES-7-Fe_3_O_4_/MIPs. With RSM, the optimal recovery conditions for theobromine and theophylline were ratio of methanol (80%) as the washing solution, methanol/HAc (8:2) eluent at pH 3, and an eluent volume of 4 mL. The practical recoveries of theobromine and theophylline in green tea were 92.27% and 87.51%, respectively, with corresponding extraction amounts of 4.87 mg·g^−1^, and 5.07 mg·g^−1^. Overall, the proposed approach with the high affinity of Fe_3_O_4_/MIPs might offer a novel method for the purification of complex isomers samples. 

## Figures and Tables

**Table 1:** The standard curve equations of theobromine and theophylline.

Analyte	Linear Equations	Correlation Coefficient
Theobromine Theophylline	*Y = 9.1044 × 10^4^ + 1.3283 × 10^4^ X* *Y = −1.0309 × 10^4^ + 6.1173 × 10^4^ X*	0.9993 0.9999

**Table 2:** Intra-day and Inter-day precisions and accuracies of theobromine and theophylline.

Analyte	Concentration (μg mL^−1^)	Intra-Day		Inter-Day	
		Recovery (%)	RSD (%)	Recovery (%)	RSD (%)
Theobromine	10	96.74	2.06	94.34	1.31
	50	92.68	3.32	91.89	3.17
	100	90.37	4.19	89.96	3.94
Theophylline	10	95.79	2.15	93.96	2.07
	50	91.62	4.10	92.27	3.92
	100	89.13	3.36	87.72	4.03

**Table 3:** Abbreviations of DES as modified solvents.

Modified Solvents	HBA	HBD	Molar Ratios	Abbreviations
DES	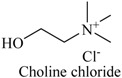	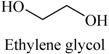		DES-1
		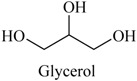		DES-2
		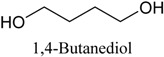		DES-3
		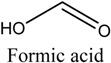	1:2	DES-4
		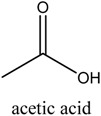		DES-5
		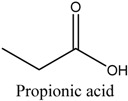		DES-6
		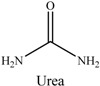		DES-7

**Table 4:** Abbreviations of ILs as modified solvents.

Modified Solvents	Cation	Anion	Molar Ratios	Abbreviations
ILs	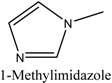	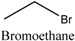	1:1	ILs-1
		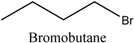		ILs-2
		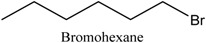		ILs-3
		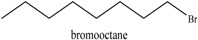		ILs-4

**Table 5:** Independent variables and their levels used for BBD.

Variables	Level		
	−1	0	1
Ratio of methanol (*X_1_*) (%)	0	50	100
PH of eluent (*X_2_*)	2	5	8
Volume of eluent (*X_3_*) (mL)	1	3.5	6
